# Hemopericardium Due to Atrioventricular Disruption After Mitral Valve Replacement With Nonemergent Left Ventricular Pseudoaneurysm Repair

**DOI:** 10.1016/j.atssr.2025.09.037

**Published:** 2025-11-05

**Authors:** Nikia T. Toomey, Zena Saleh, Jeffrey Lee, Vivek Kulkarni, Evan Carabelli, Sarah Van Dyke, Gary Cook, David Shersher, Michael Rosenbloom, Kenji Minakata

**Affiliations:** 1 Department of Surgery, Cooper University Hospital, Camden, New Jersey; 2 Department of Cardiology, Cooper University Hospital, Camden, New Jersey; 3 Division of Cardiac Surgery, Cooper University Hospital, Camden, New Jersey

## Abstract

Left ventricular (LV) pseudoaneurysm is a rare but potentially lethal complication after mitral valve (MV) repair or replacement. Typically, a perforation of the LV results in acute massive hemorrhage, pericardial effusion, and cardiac tamponade requiring emergent surgical intervention. LV pseudoaneurysms often complicate surgical intervention and result in high mortality. Alternatively, rupture may be present less acutely in the form of a well-developed pseudoaneurysm without any evidence of pericardial effusion, tamponade, or ongoing hemorrhage. This case represents a rare presentation of LV pseudoaneurysm with a challenging diagnostic course.

Left ventricular (LV) perforation after mitral valve (MV) surgery is caused most often by tissue injury in the periannular area due to intraoperative retraction with atrioventricular (AV) groove disruption or stiffening of the annulus by placement of a rigid ring prosthesis with subsequent subannular LV wall stress. It can also be associated with perioperative myocardial infarction or infective endocarditis.^1^ LV rupture after MV replacement (MVR) is classified according to 2 features: location (type 1, posterior AV groove; type 2, posterior LV at the base of the papillary muscle; and type 3, between the AV groove and papillary muscle)^2^ and timing (early or delayed).^3^

Early ruptures are those that occur while still on cardiopulmonary bypass, and mortality approaches 50% despite intraoperative identification.^4^ Delayed ruptures may present hours to months after surgery and are associated with extremely high mortality (90%).^4^ LV pseudoaneurysms represent late LV perforations that are well contained by the pericardium, pericardial adhesions, or a combination of these, which prevent free perforation, circumferential hemopericardium, tamponade, or exsanguination, and can be asymptomatic in up to 50% of cases.^1^ These usually require surgical or percutaneous intervention.^5^ We describe an LV pseudoaneurysm with hemopericardium that required definitive surgical repair.

A 68-year-old woman presented with severe mitral regurgitation at another facility. Her medical history included paroxysmal supraventricular tachycardia, hypertension, hyperlipidemia, and asthma. At the facility, she underwent MVR with a bovine pericardial valve (31-mm Mitris Resilia, Edwards Lifesciences) with posterior leaflet preservation, in a noneverting fashion, and internal suture closure of the left atrial appendage under standard median sternotomy.

Her postoperative course was complicated by surgical bleeding, for which she underwent reexploration on postoperative day 1. She was found to have a large hematoma at the base of the left atrial appendage with active oozing. This was controlled by placing an external ligation device (40-mm Atriclip, Atricure). The remainder of her index hospitalization was uneventful, and she was discharged home.

Over the course of 6 weeks postoperatively, she presented to our emergency department multiple times with chest pain and dyspnea. Multiple computed tomographic (CT) studies demonstrated hemopericardium with suggestion of recent bleeding, and transthoracic echocardiograms demonstrated a small to moderately sized circumferential pericardial effusion. During this time, she remained clinically stable.

A repeat CT scan demonstrated a 2.0-cm × 2.8-cm pseudoaneurysm arising from the LV just below the MV annulus. Initially, it appeared that a small area of contrast on the CT was possible recanalization of the left atrial appendage, because the left atrial appendage was initially closed by an internal suture; however, subsequent cardiac CT showed a clearly defined pseudoaneurysm arising from the MV annulus (Figure 1). The patient underwent further studies, including transesophageal echocardiography (TEE) (Figure 2A) and cardiac catheterization (Figure 2B), both of which supported the diagnosis of LV pseudoaneurysm.

**Figure 1:**
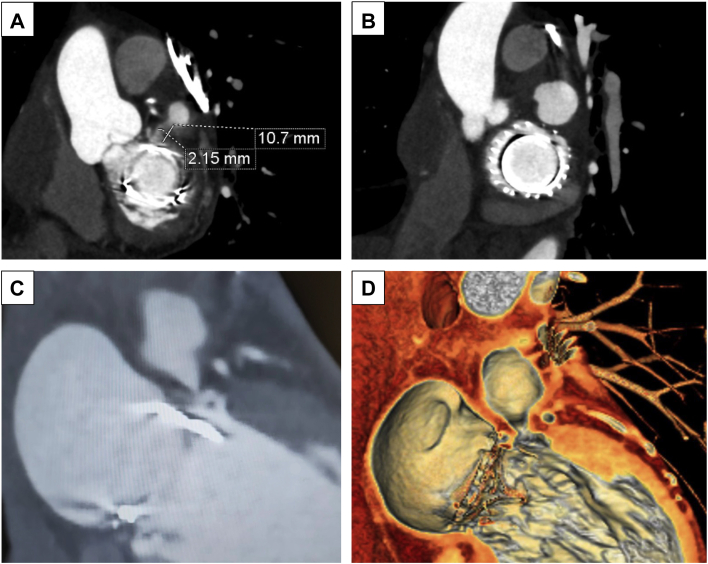
Computed tomography images show (A) a narrow communication between the left ventricle and an aneurysm sac and (B, C) an aneurysm sac adjacent to the mitral prosthesis. (D) Three-dimensional reconstruction of the aneurysm.

**Figure 2:**
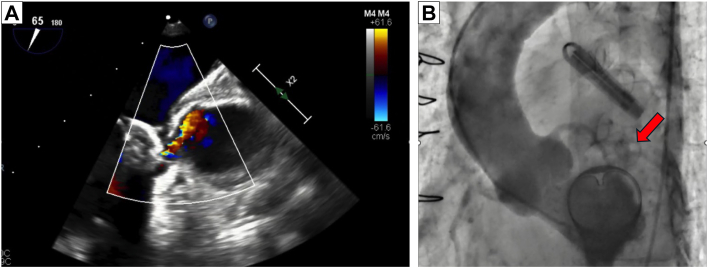
(A) Transesophageal echo image, with Doppler signal detected from the left ventricle to the aneurysm sac. (B) Left ventriculography showed contrast extravasation from the left ventricle (arrow).

We ultimately decided to perform surgery at our institution. Redo sternotomy was performed, and the patient was placed on cardiopulmonary bypass with distal ascending aortic and bicaval cannulations. Under aortic cross-clamping using del Nido cardioplegia, her MV prosthesis was exposed through a superior transseptal approach, which is our standard and offers excellent exposure. The MV prosthesis appeared intact and was removed. It was obvious that some of the posterior annular sutures were misplaced off the mitral annulus toward the left atrium. Careful inspection revealed a small perforation just a few millimeters below the mitral annulus at the 9 o’clock position in the surgeon’s view, slightly posteriorly from the posteromedial commissure (Figure 3).

**Figure 3:**
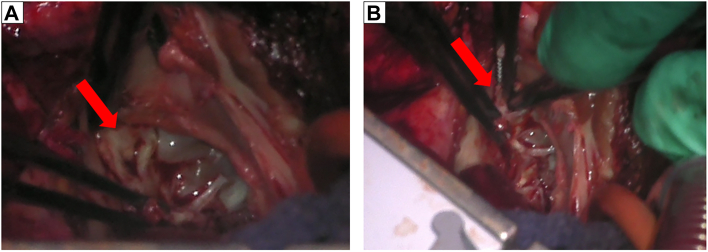
Intraoperative photographs from the headlight camera (the patient's head is on the left). (A) A definite perforation (red arrow) was identified at a subannular area, which appeared to be surrounded by healthy endothelium. (B) Forceps were inserted into the perforation to confirm the depth (red arrow).

The endothelium around the defect was normal; thus, we elected to close the defect with the valve-replacing sutures, instead of closing it with a separate patch. A new 31-mm bovine pericardial valve was implanted in the usual fashion. In total, 18 pledgetted 2-0 braided sutures were placed circumferentially on the mitral annulus with the pledgets on the ventricular side. Of note, 2 pairs of these sutures were placed deep enough to close the defect.

The patient was weaned off cardiopulmonary bypass without difficulty. The aortic cross-clamp time and the cardiopulmonary bypass time were 102 minutes and 157 minutes, respectively. Postbypass TEE demonstrated no flow signal into the pseudoaneurysm sac. Atrial fibrillation developed postoperatively, for which the patient underwent successful electrical cardioversion with TEE monitoring. TEE again demonstrated clear evidence of partial thrombosis without flow into the pseudoaneurysm sac. She was discharged to home thereafter.

At the 3-month follow-up, she was doing well without any cardiopulmonary complaints. A transthoracic echocardiogram demonstrated a well-seated mitral prosthesis with normal leaflet excursion, mean transvalvular pressure gradient of 7 mm Hg, no pericardial effusion, no residual pseudoaneurysm, and a LV ejection fraction of 0.55 to 0.60.

## Comment

The described case is a rare presentation of an LV pseudoaneurysm. Owing to the slow development of the patient’s symptoms and imaging findings, identification of the defect as an LV perforation was difficult; free perforation would result in rapid exsanguination or development of tamponade, cardiac arrest, and death. However, it appears that she had LV perforation as demonstrated by circumferential hemopericardium and early nonprogressive but persistent chest discomfort, and subsequently had prompt containment of this perforation that organized into a well-defined LV pseudoaneurysm. Our evaluations of this patient’s cardiac anatomy appear to have occurred as her pseudoaneurysm definitively developed, making the diagnosis and definitive management challenging.

Early detection of this rare entity is critical to improving the prognosis for patients with LV perforation by reducing the risk of pseudoaneurysm expansion ultimately leading to rupture. Historically, a left ventriculogram has been the primary imaging method for diagnosing LV pseudoaneurysms^6^; however, cardiac CT, magnetic resonance imaging, and echocardiography are excellent noninvasive diagnostic tools that collectively offer good diagnostic quality. Transthoracic echocardiogram has a 25% to 31% sensitivity for LV pseudoaneurysms, and TEE improves sensitivity to 75%.^7^ Cardiac magnetic resonance imaging offers sensitivity and specificity >90% for detecting LV pseudoaneurysms,^8^ but is associated with higher costs, and interpretation requires greater expertise, which may limit its use. The use of echocardiography and cardiac CT offers a cost-effective multimodality approach for evaluating cardiac anatomy postoperatively to identify abnormalities, such as pseudoaneurysms, that require intervention.

Although this is an extremely rare presentation of an already rare entity, this case highlights that absence of catastrophic clinical deterioration does not necessarily exclude the presence of potentially devastating cardiac structural defects after MV surgery. It emphasizes the need for a high index of suspicion for LV perforation or rupture, especially in a patient with persistent pericardial effusion.
